# Within-Person Variation in Nutrient Intakes across Populations and Settings: Implications for the Use of External Estimates in Modeling Usual Nutrient Intake Distributions

**DOI:** 10.1093/advances/nmaa114

**Published:** 2020-10-15

**Authors:** Caitlin D French, Joanne E Arsenault, Charles D Arnold, Demewoz Haile, Hanqi Luo, Kevin W Dodd, Stephen A Vosti, Carolyn M Slupsky, Reina Engle-Stone, The Variance Components of Nutrient Intakes Data Working Group, Reina Engle-Stone, Caitlin D French, Joanne E Arsenault, Charles D Arnold, Demewoz Haile, Doris Wiesmann, Yves Martin-Prevel, Inge D Brouwer, Melissa C Daniels, Christine Delisle Nyström, Marie Löf, Alex Ndjebayi, Cristina Palacios, Lukkamol Prapkree, Amanda Palmer, Bess L Caswell, Kenneth Hn Brown, Georgn Lietz, Marjorien Haskell, Jody Miller

**Affiliations:** Department of Nutrition, University of California, Davis, Davis, CA, USA; Institute for Global Nutrition, University of California, Davis, CA, USA; Intake–Center for Dietary Assessment, FHI Solutions, Washington, DC, USA; Institute for Global Nutrition, University of California, Davis, CA, USA; Department of Nutrition, University of California, Davis, Davis, CA, USA; Institute for Global Nutrition, University of California, Davis, CA, USA; Department of Nutrition, University of California, Davis, Davis, CA, USA; Institute for Global Nutrition, University of California, Davis, CA, USA; National Cancer Institute, National Institutes of Health, Rockville, MD, USA; Department of Agricultural and Resource Economics, University of California, Davis, CA, USA; Department of Nutrition, University of California, Davis, Davis, CA, USA; Department of Food Science and Technology, University of California, Davis, Davis, CA, USA; Department of Nutrition, University of California, Davis, Davis, CA, USA; Institute for Global Nutrition, University of California, Davis, CA, USA; Nutripass, University of Montpellier, Institut de Recherche pour le Développement, Montpellier SupAgro, Montpellier, France; Division of Human Nutrition and Health, Wageningen University, Wageningen, Netherlands; Department of Nutrition, University of North Carolina, Chapel Hill, NC, USA; Department of Biosciences and Nutrition, Karolinska Institutet, Stockholm, Sweden; Department of Biosciences and Nutrition, Karolinska Institutet, Stockholm, Sweden; Helen Keller International, Yaoundé, Cameroon; Department of Dietetics and Nutrition, Florida International University, Miami, FL, USA; Department of Dietetics and Nutrition, Florida International University, Miami, FL, USA; Department of International Health, Johns Hopkins Bloomberg School of Public Health, Baltimore, MD, USA; Department of Nutrition and Institute for Global Nutrition, University of California, Davis, Davis, CA, USA; Department of Nutrition and Institute for Global Nutrition, University of California, Davis, Davis, CA, USA; Human Nutrition Research Centre, Population Health Sciences Institute, Newcastle University, Newcastle upon Tyne, UK; Department of Nutrition and Institute for Global Nutrition, University of California, Davis, Davis, CA, USA; Department of Nutrition and Institute for Global Nutrition, University of California, Davis, Davis, CA, USA

**Keywords:** dietary assessment, measurement error, within-individual variation, variance components, variance ratio, micronutrients

## Abstract

Determining the proportion of a population at risk of inadequate or excessive nutrient intake is a crucial step in planning and managing nutrition intervention programs. Multiple days of 24-h dietary intake data per subject allow for adjustment of modeled usual nutrient intake distributions for the proportion of total variance in intake attributable to within-individual variation (WIV:total). When only single-day dietary data are available, an external adjustment factor can be used; however, WIV:total may vary by population, and use of incorrect WIV:total ratios may influence the accuracy of prevalence estimates and subsequent program impacts. WIV:total values were compiled from publications and from reanalyses of existing datasets to describe variation in WIV:total across populations and settings. The potential impact of variation in external WIV:total on estimates of prevalence of inadequacy was assessed through simulation analyses using the National Cancer Institute 1-d method. WIV:total values were extracted from 40 publications from 24 countries, and additional values were calculated from 15 datasets from 12 nations. Wide variation in WIV:total (from 0.02 to 1.00) was observed in publications and reanalyses. Few patterns by population characteristics were apparent, but WIV:total varied by age in children (< vs. >1 y) and between rural and urban settings. Simulation analyses indicated that estimates of the prevalence of inadequate intake are sensitive to the selected ratio in some cases. Selection of an external WIV:total estimate should consider comparability between the reference and primary studies with regard to population characteristics, study design, and statistical methods. Given wide variation in observed ratios with few discernible patterns, the collection of ≥2 days of intake data in at least a representative subsample in population dietary studies is strongly encouraged. In the case of single-day dietary studies, sensitivity analyses are recommended to determine the robustness of prevalence estimates to changes in the variance ratio.

## Teaser

Variation in within-person variance component ratios of nutrient intakes was reviewed to assess statistical implications for studies analyzing single-day 24-h dietary recall or food record data.

## Introduction

Assessing population nutrient intakes is a crucial step in planning and evaluating interventions to address nutrient inadequacies or excessive intakes and related health problems. Determining the proportion of a population with inadequate or excessive intake of nutrients requires knowledge of the distribution of usual (habitual) intakes in comparison to theoretical requirements (1). While the distribution of physiological nutrient requirements for a population is usually unknown, methods exist to approximate the prevalence of nutrient inadequacy by making assumptions about the distribution of requirements (1, 2). The Estimated Average Requirement (EAR) cut-point method uses the proportion of the population whose intake falls below the EAR for a nutrient to estimate the prevalence of inadequate intakes (3). This method is subject to several key assumptions, including that *1*) usual intakes are independent of nutrient requirements, *2*) the distribution of requirements is approximately symmetrical, *3*) the variance in usual intakes is greater than that of requirements, and *4*) the actual prevalence of inadequate intake is neither very low (<8 to 10%) nor very high (>90 to 92%) (3).

Twenty-four-hour dietary recalls (24HRs) or weighed food records are commonly used to gather data on food consumption that can be converted to daily nutrient intakes. Although food and nutrient intakes captured on any single day do not reflect long-term average, or usual, intake, due in large part to day-to-day variation in food consumption for a given individual, collection of 1 d of recall data on enough individuals (representing both weekdays and weekends) may accurately estimate the average intake of a population, assuming that the recalls are unbiased and that the measurement error around usual intake is random. However, even in this case, using unadjusted, single-day estimates to obtain population distributions of usual intakes and calculate prevalence of inadequacy (e.g., using the EAR cut-point method) is inappropriate because the variance in these distributions will be inflated relative to the usual intake distribution due to the contribution of within-individual variation (WIV) (4). To avoid this, it is recommended to collect information on dietary intake on >1 d from at least a representative subset of study participants and apply appropriate statistical methods to model usual intake distributions (3, 4). Current methods, such as those developed by the National Cancer Institute (NCI) (5) and Iowa State University (6), decompose total variation in transformed nutrient intake into WIV and between-individual variation (BIV). After computing WIV and BIV, the distribution of usual intakes can be modeled reflecting only the BIV component (4).

Due to limited resources or feasibility constraints at the time of data collection, multiple days of dietary intake data may not be available at the time of analysis, precluding the separation of variance components from the observed data. In these instances, an external estimate of the fraction of total variance attributable to WIV (WIV:total) may be used in conjunction with the total variance estimate for the study population to estimate the usual intake distribution (7). There is currently no formal guidance on how to select appropriate external variance ratios. It is conceivable that WIV (and BIV) of nutrient intakes may vary with different dietary customs, food security, seasonal agricultural patterns, etc., and by nutrient. In such cases, using an external adjustment factor from a population for which the WIV:total differs substantially from the study population may lead to inaccurate assessments of population usual nutrient intake distributions. Sensitivity analyses have shown, for some nutrients and populations, that the choice of an external WIV:total value for analyzing single-recall datasets can influence estimates of prevalence of nutrient inadequacy to varying degrees, depending on the extent to which the external value deviates from the “true” variance ratio—that is, that observed from data from the same population including ≥2 days of intake on at least a subsample (7–9). However, for many nutrients, in particular in low- and middle-income countries, the degree to which WIV:total varies by population and setting and how estimated prevalence of inadequacy may be influenced by this variation is not well understood. The present study therefore aims to *1*) describe variation in variance component ratios across a wide range of global contexts and provide a database of WIV:BIV and WIV:total estimates for a range of populations that may be consulted for selection of external adjustment factors and *2*) assess the impact of potential variation in these ratios on the prevalence of inadequate intake in models of usual nutrient intake distributions using single-day nutrient intake data. Last, we propose an approach for selecting and applying external variance estimates for modeling usual nutrient intake distributions.

## Methods

### Literature review and compilation of variance component estimates from existing datasets

#### Literature review

To describe variance component ratios from diverse studies, we first conducted a literature review of studies that reported variance ratios for ≥1 nutrient. We identified studies that included ≥2 nonconsecutive (or ≥3 consecutive) days of dietary intake data collected by 24HRs or food records and that reported variance components of micronutrient intakes in the related publication or report. We reviewed 5 micronutrients of public health concern in low- and middle-income countries (10–14), including vitamin A, folate, vitamin B-12, iron, and zinc. Additional data on total energy intake, retinol, vitamin C, thiamin, and carotenoids were compiled as secondary nutrients of interest, as available. Searches were conducted using the National Center for Biotechnology Information, Google, the Dietary Assessment Calibration/Validation Register (https://epi.grants.cancer.gov/dacv/) (15), the Global Individual Food Consumption data tool (http://www.fao.org/gift-individual-food-consumption/en/) (16), and snowball searches. Variance component ratios were extracted from publications and reports. Here we report these values as *1*) the proportion of total variance attributable to WIV (WIV:total), and *2*) the ratio of within- to between-individual variance (WIV:BIV). When ≥1 of these was not reported in the publication, reported values were converted to the statistics of interest, if possible. For example, when ratios of CVs of WIV and BIV were reported in the publication, the CV ratio was squared to obtain the ratio of variances. The types of ratios reported in publications and calculations used to convert between them are described in **Tables 1** and **2**, respectively. When reported ratios were calculated for data both before and after statistical transformations, the most appropriate ratio was chosen based on which better complied with model assumptions as described in the publication. Descriptive statistics were calculated for each nutrient and subpopulation to describe the range and distributions of variance component ratios using RStudio version 1.2.1335 software (RStudio, Inc.).

**TABLE 1:** Formulas for different types of variance ratios^1^

Description	Formula
Within- to between-individual CVs (*R_CV_*)	$\frac{{C{V_w}}}{{C{V_b}}}$
Within- to between-individual variance (α)	$\frac{{{s_w}^2}}{{{s_b}^2}}$
Within-individual to total variance^2^ (β)	$\frac{{{s_w}^2}}{{( {{s_b}^2 + {s_w}^2} )}}$

1 *CV_b_*, between-individual CV; *CV_w_*, within-individual CV; *s_b_*^2^, between-individual variance; *s_w_*^2^, within-individual variance.

2 For studies including covariates or other sources of variation in the analyses from which variance ratios were calculated, total variance refers to residual variance after accounting for the contribution of these variables.

**TABLE 2:** Calculations used to convert among types of variance ratios^1^

Ratios to convert	Formula
*R_CV_* → ${\rm{\alpha }}$	${\rm{\alpha }} = {( {{R_{CV}}} )^2}$
α → β	${\rm{\beta }} = \frac{{\rm{\alpha }}}{{( {1 + {\rm{\alpha }}} )}}$
β → α	${\rm{\alpha }} = \frac{{\rm{\beta }}}{{( {1 - {\rm{\beta }}} )}}$

1 *R_CV_*, ratio of within-to between-individual CVs; α, ratio of within- to between-individual variance; β, ratio of within-individual to total variance.

#### Reanalysis of existing datasets

To expand the available data, and after observing wide variation in methods of calculating and reporting variance components, we reanalyzed existing datasets to calculate variance components using a consistent method. Authors of studies that calculated nutrient intakes from multiple days of dietary recall/record data but did not report variance component values in their publications were contacted and invited to participate as collaborators to reanalyze their data to generate variance component estimates. Collaborators who agreed were provided with analytical code compatible with R or SAS software (available on the Open Science Framework at https://osf.io/wyvug/) along with instructions detailing how to structure the datasets and modify the code to run on each dataset. In addition, several available or shared datasets were analyzed by authors of this manuscript (JEA, CDA, DH, and CDF). As available, the same micronutrients of interest as above, including vitamin A, folate, vitamin B-12, iron, and zinc, were analyzed. Since it was convenient to analyze additional nutrients from the same code, ratios were also calculated for total energy, retinol, thiamin, phytate, and synthetic folic acid intakes, as available.

Datasets were first divided into subsets based on age, sex, physiological status (i.e., pregnancy and lactation), season, and/or rural versus urban setting, according to the original study's analysis or where sample sizes allowed. For 1 dataset (17), separate analyses were conducted including or excluding iron intake from cooking pots for comparability with other studies considering only intrinsic iron in food. Variance components were then calculated for each subset using a linear mixed model with restricted maximum likelihood estimation [PROC MIXED procedure in SAS or the lmer function of the lme4 package in R (18)] based on the NCI approach to variance component estimation (5). Data were transformed using an iterative Box-Cox transformation based on normality of the residuals. The mixed model was then fit with the transformed nutrient intakes as the response variable, recall sequence as a fixed-effect predictor, and subject as a random effect. When available in the dataset, an additional fixed-effect variable was included to indicate whether or not the recall period included a weekend day. In these cases, “weekend” was defined by each collaborator as appropriate for the particular study population. WIV (expressed as within-individual variance) was defined as the mean square error for the model, and BIV (as between-individual variance) used the mean square error for the person-specific random effect, after accounting for the effects of weekend (when available) and recall sequence. From these variance estimates, WIV:BIV and WIV:total were then calculated using formulas displayed in Table 1.

For 1 national survey in Cameroon (19), the NCI MIXTRAN SAS macro (20) was used to calculate variance ratios to allow for survey weights to be incorporated. As a sensitivity analysis to observe the effect of covariates on variance component estimates in this dataset, 3 models were generated for each nutrient: *1*) accounting only for weekend and interview sequence (unadjusted model), *2*) including survey stratum as an additional covariate (minimally adjusted model), and *3*) including all relevant covariates (fully adjusted model). For the fully adjusted models in women and children, additional covariates included interviewer, translator, age, caregiver educational level, and socioeconomic status. In addition, child sex and breastfeeding status were included as covariates in models in children, while physiological status (pregnant or lactating) and whether the day of intake was a feast day were included as covariates in models in women.

### Simulation analysis

To assess the effects of variability in assumed WIV:total on estimated prevalence of inadequate intake when analyzing 1-d data, the EAR cut-point method was applied to usual nutrient intake distributions modeled using the NCI 1-d method (9). Two datasets representing different geographic settings and having multiple days of dietary intake data had previously been analyzed by 2 authors of this manuscript (RE-S, JEA) and were available for this analysis. These included 24HRs from the aforementioned national survey conducted in Cameroon (19) and weighed food records from a survey in 2 rural districts in Bangladesh (21). For the present analysis, the Cameroon sample included 24HR data on 537 nonpregnant, nonlactating (NPNL) women of reproductive age (WRA), and the Bangladesh sample provided food record data on 463 NPNL or minimally lactating (breastfeeding 2- to 3-y-old children) WRA. The EARs used as cutoffs for inadequacy were 320 μg/d for folate (22), 500 μg retinol activity equivalents/d for vitamin A (23), and 6 mg/d for zinc (24). The EAR used for zinc as determined by the International Zinc Nutrition Consultative Group assumes a mixed or refined vegetarian diet (24). The first day of intake only served as the single-day datasets for the simulations, while the full datasets allowed for the calculation of the “true” or expected WIV:total from these populations. Simulations of intake distributions for folate, vitamin A, and zinc in WRA (19–50 y) were performed using the single-day intake data and hypothetical external WIV:total ratios covering a wide range of possible values (0.05, 0.10…0.95, 0.99). As a sensitivity analysis, the effect of varying ratios on uncertainty in the prevalence estimates was investigated for the Cameroon dataset using balance repeated replication with 48 sets of replicates and a Fay coefficient of 0.7 to calculate SEs on a subset of simulations (25).

To compare the simulations with results that would be observed with multiple-day data, WIV:total values for these samples were calculated from the full datasets for the population samples specified above, which included a second dietary recall or record on at least a subset of the population. For the Bangladesh data, the linear mixed-model process described in the preceding section for reanalysis of existing datasets was applied for calculation of variance components. For the Cameroon data, variance components were calculated using the NCI MIXTRAN macro (5) conducted in SAS version 9.4 (SAS Institute) to account for the complex sampling design used in this study. These ratios were applied to the NCI 1-d method to produce prevalence estimates for comparison to the simulations with hypothetical variance ratios. For comparability between the Cameroon and Bangladesh analyses, we used minimally adjusted models accounting for interview sequence and, in the case of Cameroon, weekend and survey stratum, with the purpose of demonstrating the effect of shifts in the variance ratio rather than to provide a best estimate of real prevalence, which may be sensitive to other covariates (26, 27).

## Results

### Characteristics of studies reporting on variance components or used for reanalysis

Forty publications and reports representing 24 countries that described studies collecting multiple days of dietary intake data and reported variance component values for nutrient intakes were included in the literature review. While ≥1 estimates were available from a number of countries representing Asia (28–41), Africa (38, 42, 43), Europe (7, 35, 44–50), and Latin America (38, 51–54), the vast majority of available variance component data represented North American populations (7, 8, 40, 47, 55–65). In particular, a plethora of variance component ratios are available from 2 nationally representative surveys from the United States, the Continuing Survey of Food Intakes by Individuals (CSFII) and NHANES, including from different survey years and a wide range of age and sex subgroups (7, 8, 47, 57, 62, 65).

Published studies reporting on variance components used a range of study designs and sampling procedures (**Table 3**). Dietary assessment methods included 24HRs, weighed or nonweighed food records, or a combination of these and included consecutive and/or nonconsecutive days. Studies varied in the reported number of days of data (range: 2–28 d) and the time interval between measurements (i.e., consecutive to 1 y).

Components of variation (WIV and BIV) were reported in publications in several forms, including within-individual and between-individual variances, SDs, or CVs. Ratios of these were also reported, as well as “variance component ratios” (i.e., the proportion of total variance attributable to WIV and/or BIV). In addition, a number of different statistical methodologies were used in the calculation of variance components. Approaches to data transformation ranged from no transformation to power, log, or Box-Cox transformations and/or multistep transformation processes (6). Transformations were applied to either all data or select nutrients based on their effect on statistical assumptions.

After 4 external collaborators shared or reanalyzed data from existing studies (17, 66-76) and several datasets (21, 26,75, 77– 84) were analyzed by some of the authors (JEA, CDF, DH, and CDA), variance ratio data were available from 15 studies from 12 countries or territories. Study populations in these datasets included women and children of varying age ranges. Fourteen of the 15 datasets included intake data for vitamin B-12, dietary folate, iron, thiamin, vitamin A, and zinc, while limited information was available for phytate, folic acid, and retinol (2, 3, and 6 studies had available data for these nutrients, respectively). Characteristics of all included studies are listed in Table 3.

### Variation in within-individual to total variance ratios

**FIGURE 1:**
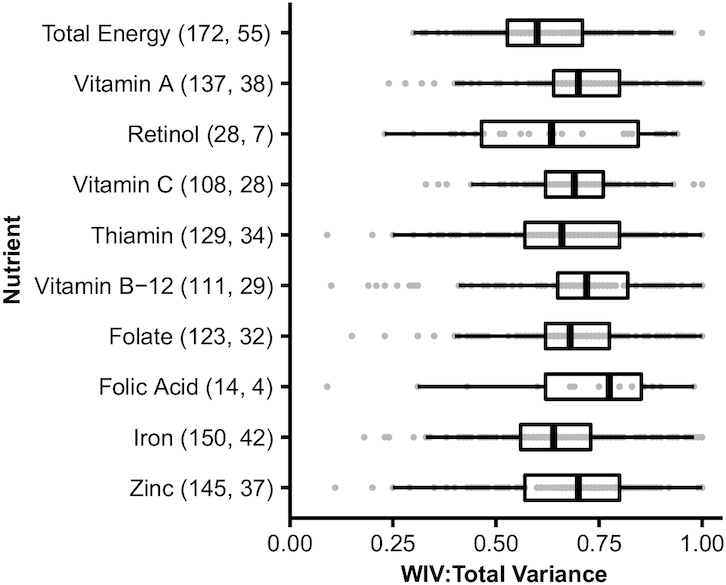
Ratios of within-individual to total variance in nutrient intakes from publications or reanalyzed datasets. The total number of ratio estimates (points) and studies, respectively, represented by each boxplot are shown in parentheses on the vertical axis. Data include analyses on overlapping populations when combined and disaggregated analyses were performed by season, age, sex, or physiological status, or including and excluding the contribution of iron intake from cooking pots. Ratios for analyses including intake from supplements are not shown. “Folic acid” refers to the synthetic form found in fortified foods, while “folate” refers to natural dietary forms or dietary folate equivalents. WIV:total variance, ratio of within-individual to total variance.

**FIGURE 2:**
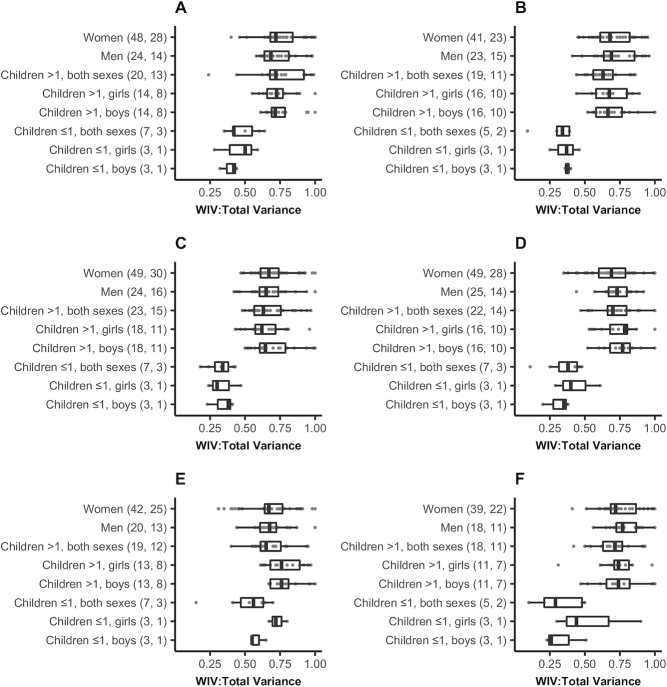
Ratios of within-individual to total variance in intakes of vitamin A (A), thiamin (B), iron (C), zinc (D), folate (E), and vitamin B-12 (F) from publications or reanalyzed datasets, by age and sex group. The total number of ratio estimates (points) and studies, respectively, represented by each boxplot are shown in parentheses on the vertical axis. Women included nonpregnant, nonlactating; pregnant; lactating; or mixed populations. Children >1 y included age ranges falling between 1 and 20 y of age. Children ≤1 y included ages up to 13 mo in some cases. Ratios for other age-sex groupings are not shown due to small numbers of available estimates. Data include analyses on overlapping populations when combined and disaggregated analyses were performed by season, age, or physiological status or including and excluding the contribution of iron intake from cooking pots. WIV:total variance, ratio of within-individual to total variance.

WIV:total ratios extracted from publications varied widely overall (from 0.02 to 1.00). Using a consistent statistical method, ranges of WIV:total ratios from reanalyzed datasets exhibited similarly wide variation (from 0.09 to 1.00, excluding 1 ratio of 1.04 due to an implausible negative variance). Excluding ratios derived from analyses considering intake from supplements, which generally resulted in lower ratios than those considering only diet, distributions of WIV:total ratios by micronutrient revealed some variation in median ratios by nutrient, but even wider variation in ratios within nutrients (**Figure 1**). WIV:total ratios of vitamin C intakes displayed the narrowest range (0.33–1.00), while WIV:total of thiamin intakes exhibited the widest range (0.09–1.00). Since dietary intake patterns and resulting variance ratios may vary by age and sex, distributions of WIV:total were also assessed by age and sex group (**Figure 2**). Distributions suggest generally lower ratios among children <1 y of age compared with other age groups. No clear patterns emerged by age or sex among populations >1 y of age. Further comparisons among women by pregnancy or lactation status were not possible for any micronutrients due to a limited number of studies assessing intake during these periods.

**TABLE 3:** Characteristics of included studies^1^

Region	Country or territory	Study description	Total sample size^2^	Populations studied	Repeats on all subjects or subsample	No. of days among those with repeats^3^	Dietary assessment method	Nutrients included^4^	Year(s) of dietary data collection	Reference(s)
Literature review										
Africa	Burkina Faso	Dietary validation study among adolescents in a primarily urban district in central Burkina Faso	237	Adolescents	Subsample	2	24HR, food records	Vitamin A, vitamin C, thiamin, vitamin B-12, folate, iron, zinc	2019	Arsenault et al. (2020) (43)
Africa	Egypt	Cohort (Collaborative Research Support Program on Food Intake and Human Function, Egypt)^5^	NR	Women	NR	NR	NR	Total energy	NR	Nyambose et al. (2002) (38)
Africa	Kenya	Cohort (Collaborative Research Support Program on Food Intake and Human Function, Kenya)^5^	NR	Women	NR	NR	NR	Total energy	NR	Nyambose et al. (2002) (38)
Africa	Malawi	Dietary survey among pregnant women in a rural area in central Malawi	184	Women	All or most	2–12	Food records	Total energy, vitamin A, vitamin C, vitamin B-12, folate, iron, zinc	1988–1991	Nyambose et al. (2002) (38)
Africa	Uganda	Nationally representative survey (2008 Uganda Food Consumption Survey)	1467	Women, children	Subsample	2	24HR	Vitamin A, iron, zinc	2008	Ethiopian National Food Consumption Survey Report (2013) (42, 85)
Asia	Bangladesh	Observational study in a nonrandom sample reflecting range of socioeconomic status (Matlab study)	834	Women, men, children, adolescents	All or most	6	Food records	Total energy	1977–1978	Torres et al. (1990) (30)
Asia	India	Dietary survey in south India (International Crops Research Institute for Semi-Arid Tropics Survey)	368	General population (combined age and sex groups)	All or most	4	NR	Total energy	1976–1977	Bhargava (1992) (28)
Asia	India	Dietary validation study among male tobacco users and household members in rural communities in Gujarat and Kerala	120	Women, men	All or most	8	24HR	Total energy, B-carotene, vitamin C, thiamin, iron, zinc	1993–1994	Hebert et al. (2000) (36)
Asia	Indonesia	Observational dietary study among pregnant women in rural Indonesia	743	Women	All or most	9	Food records	Total energy, vitamin A	1982–1984	Reported in Nyambose, et al. (2002) (38), originally from Launer et al. (1991)^6^ (86)
Asia	Indonesia	Representative 2-stage cluster longitudinal survey of pregnant women in a mostly rural district	451	Women	All or most	6	24HR	Total energy, vitamin A, vitamin C, thiamin, iron	1996–1998	Persson et al. (2001) (39)
Asia	Japan	Dietary validation study in 2 rural towns	113	Women, men	All or most	12	Food records	Total energy, vitamin A, thiamin, vitamin B-12, folate, iron, zinc	1996–1997	Tsubota-Utsugi et al. (2013) (31)
Asia	Japan	Dietary validation study among dietitians	80	Women	All or most	28	Food records	Total energy, vitamin A, vitamin C, iron, zinc	1996–1997	Imaeda et al. (2013) (37); Tokudome et al. (2002) (41)
Asia	Japan	Observational dietary survey in 4 areas in Japan	242	Women, men	All or most	16	Food records	Total energy, vitamin A, carotenoids, vitamin C, thiamin, vitamin B-12, folate, iron, zinc	2002–2003	Fukumoto et al. (2013) (34)
Asia	Japan	Pre-intervention data from a randomized controlled trial	56	Men	All or most	7	Food records	Total energy, vitamin A, vitamin C, thiamin, vitamin B-12, folate, iron, zinc	2013–2015	Taguchi et al. (2017) (32)
Asia	Japan	National nutrition survey	181	Men	All or most	7	Food records	Total energy	1963	Liu et al. (1978) (33), Tillotson et al. (1973) (87)
Asia	Philippines	Nationally representative survey (Filipino National Food Consumption Survey 2003)	1374	Children	NR	2	Combination	Iron	2003	Gibbs et al. (2014) (35)
Asia	Philippines	Household survey randomly sampled to represent a range of sugar production access (88)	2047	General population (combined age sex groups)	All or most	4	24HR	Total energy	1984–1985	Bhargava (1992) (28)
Asia	Taiwan	FFQ validation study among nutrition major students	69	Women, men	All or most	10–15	Food records	Total energy, vitamin A, vitamin C, thiamin, iron	NR	Chang et al. (2001) (29)
EasternEurope/Asia	Russia	Nationally representative survey (Russia Longitudinal Monitoring Survey 1996)	1413	Adolescents	All or most	2	24HR	Total energy, vitamin C, thiamin, iron	1996	Jahns et al. (2004) (47)
EasternEurope/Asia	Russia	Nationally representative survey (Russia Longitudinal Monitoring Survey 2000)	103	Adolescents	All or most	2	24HR	Vitamin C	2000	Jahns et al. (2005) (7)
Europe	Belgium	Multistage cluster survey of Flemish schools (Flemish Preschool Dietary Survey 2002–2003)	661	Children	All or most	3	Food records	Total energy, vitamin C, thiamin, iron, zinc	2002–2003	Huybrechts et al. (2008) (45)
Europe	England/United Kingdom	Secondary analysis of multiple studies of varying study designs	741	Women, men, adolescents, children	All or most	7–28	Food records	Total energy, retinol, carotene, vitamin C, thiamin, vitamin B-12, folate, iron, zinc	1968–1984	Nelson et al. (1989) (50)
Europe	England	Community (dietary validation) study of children attending health clinics	72	Children	All or most	5	Food records	Total energy, vitamin A, vitamin C, iron, zinc	NR	Lanigan et al. (2004) (49)
Europe	Finland	Dietary validation study (Helsinki Diet Methodology Study)	162	Men	All or most	24	Food records	Total energy, vitamin A, vitamin C, thiamin	1984	Hartman et al. (1990) (44)
Europe	Germany	Cohort study in Potsdam (EPIC)	134	Women and men	All or most	10–12	24HR	Total energy	1994	Kroke et al. (1999) (46)
Europe	Greece	National survey including a random stratified sample in 3 counties	1794	Adolescents, children	All or most	3	Food records	Total energy, vitamin A, vitamin C, iron	NR	Roma-Giannikou et al. (1997) (48)
North America	Canada	Observational dietary study among Toronto sales and office staff (Canadian National Heart Lung Blood Institute Nutrition Data System)	60	Women, men	All or most	6	24HR	Total energy, vitamin C, thiamin, iron	1977	Beaton et al. (1983) (60)
North America	Canada	Nationally representative survey (Food Habits of Canadians Study 1997–98)	1541	Women, men	Subsample	2	24HR	Total energy, vitamin C, folate, iron	1997–1998	Palaniappan et al. (2003) (55)
North America	Canada	Nationally representative survey (2015 Canadian Community Health Survey)	20,487	Women, adolescents, children	Subsample	2	24HR	Folate	2015	Davis et al. (2019) (58)
North America	Mexico	Random, multistage sample survey of urban areas of Mexico	1063	Women, men, adolescents, children	All or most	3	24HR	Total energy, vitamin A, vitamin C, folate, iron, zinc	2012	Shamah-Levy et al. (2016) (53)
North America	Mexico	Cohort (Collaborative Research Support Program on Food Intake and Human Function, Mexico)^5^	NR	Women	NR	NR	NR	Total energy	NR	Nyambose et al. (2002) (38)
North America	United States	Nationally representative survey (CSFII 1996)	645	Adolescents	All or most	2	24HR	Total energy, vitamin C, thiamin, iron	1996	Jahns et al. (2004) (47); Jahns et al. (2005) (7)
North America	United States	Randomized controlled trial of calcium supplementation	142	Women	All or most	19	Food records	Total energy, vitamin A, vitamin C, thiamin, vitamin B-12, folate, iron, zinc	Between 1978 and 1982	Sempos et al. (1985) (56)
North America	United States	Nested random sample within cohort of senior citizens in Albuquerque	50	Women, men	All or most	6	Food records	Total energy, vitamin A, vitamin C, thiamin, vitamin B-12, folate, iron, zinc	1980–1981	Hunt et al. (1983) (63)
North America	United States	Cohort of senior citizens in south-central Connecticut	220	Women, men	All or most	4	24HR	Total energy, vitamin A, vitamin C, thiamin, vitamin B-12, folate, iron, zinc	1982–1985	McAvay and Rodin (1988) (64)
North America	United States	FFQ validation study in low-income, innercity state schools	109	Adolescents	All or most	4	24HR	Total energy, vitamin C, iron	1993–1994	Field et al. (1999) (61)
North America	United States	Nationally representative survey (NHANES 2002)	NR	Women, men, adolescents, children	All or most	2	24HR	Total energy, vitamin A, vitamin C, thiamin, vitamin B-12, folate, iron, zinc	2002	Goldman (2005) (62)
North America	United States	Nationally representative survey (NHANES 2003–2008)	18,424	Women, men, adolescents	All or most	2	24HR	Vitamin A, vitamin C, zinc	2003–2008	Brandt (2012) (8)
North America	United States	Nationally representative survey (NHANES 2007–2008)	7654	Women, men, children	All or most	2	24HR	Total energy, vitamin A, carotenoids, vitamin C, thiamin, vitamin B-12, folate, iron, zinc	2007–2008	Willett (2013) (57)
North America	United States	Nationally representative survey (NHANES 2007–2010)	3473	Adolescents, children	All or most	2	24HR	Total energy, vitamin A, vitamin C, thiamin, vitamin B-12, folate, iron, zinc	2007–2010	Ollberding et al. (2014) (65)
North America	United States	Randomized controlled trial of prenatal supplementation (Prenatal Project)	225	Women	All or most	2–4	24HR	Total energy	Between 1969 and 1976	Rush and Kristal (1982) (40)
North America	United States	Cohort study of nurses (Nurses’ Health Study)	173	Women	All or most	28	Food records	Total energy, vitamin A, vitamin C, folate, iron, zinc	1980–1981	Willett (2013) (57), Willett et al. (1985) (89)
North America	United States	Cohort of men of Japanese ancestry	318	Men	All or most	7	Food records	Total energy	1967–1970	Liu et al. (1978) (33), Tillotson et al. (1973) (87)
North America	United States	Randomized controlled clinical trial of protein supplementation among overweight and obese individuals	73	Women, men	All or most	15	24HR	Total energy, vitamin A, B-carotene, vitamin C, thiamin, vitamin B-12, folate, folic acid, iron, zinc	NR	Stote et al. (2011) (59)
South America	Brazil	Representative population-based survey in São Paulo	511	Women, men	Subsample	2–4	24HR	Total energy, vitamin A, vitamin C, thiamin, vitamin B-12, folate, iron, zinc	2007	Morimoto et al. (2011) (51)
South America	Ecuador	Small community survey (nonrandomized sample representing approximately 40% of residents)	149	Women, men, adolescents, children	All or most	2–6	24HR	Total energy, vitamin A, vitamin C, thiamin, vitamin B-12, folate, iron, zinc	1994	Berti and Leonard (1998) (54)
South America	Peru	Longitudinal dietary survey in a peri-urban community	124	Children	All or most	5 per 4 mo or 16 per 12 mo^7^	Combination	Total energy	1982–1984	Piwoz et al. (1994) (52)
Reanalyzed datasets										
Africa	Burkina Faso	Dietary survey in 2 urban districts (WDDP Burkina Faso 1)	178	Women	All or most	3	24HR	Total energy, vitamin A, thiamin, vitamin B-12, folate, iron, zinc	2006	Martin-Prevel et al. (2015) (66), Becquey et al. (2009) (67)^8^
Africa	Burkina Faso	Multistage cluster survey in 2 provinces (WDDP Burkina Faso 2)	960	Women, children	Subsample	2	24HR	Total energy, vitamin A, thiamin, vitamin B-12, folate, iron, zinc	2010	Arsenault et al. (2014) (77, 78)
Africa	Cameroon	Nationally representative cluster survey	1774	Women, children	Subsample	2	24HR	Vitamin A, vitamin B-12, folate, iron, zinc	2009	Engle-Stone et al. (2012) (19)
Africa	Lesotho	Pilot survey in a district with high prevalence of malnutrition	268	Women, children	Subsample	2	24HR	Total energy, vitamin A, thiamin, vitamin B-12, folate, iron, zinc	2009	Wiesmann et al. (2012) (17)
Africa	Mali	Multistage cluster survey (WDDP Mali)	102	Women	All or most	2	24HR	Total energy, vitamin A, thiamin, vitamin B-12, folate, iron, zinc	2007	Kennedy et al. (2009) (68)
Africa	Mozambique	Baseline survey for cluster-randomized trial (WDDP Mozambique)	254	Women	Subsample	2	24HR	Total energy, vitamin A, thiamin, vitamin B-12, folate, iron, zinc	2006	Hotz et al. (2012), 1 (80)
Africa	Uganda	Multistage cluster survey in 3 rural regions (WDDP Uganda 1)	869	Women, children	Subsample	2	24HR	Total energy, vitamin A, retinol, thiamin, vitamin B-12, folate, folic acid, iron, zinc	2007	Hotz et al. (2012), 2 (79, 81)^8^
Africa	Zambia	Baseline data from cluster-randomized controlled trial of biofortified maize	202	Children	All or most	Up to 7	24HR	Total energy, vitamin A, retinol, thiamin, vitamin B-12, folate, folic acid, iron, zinc	2012–2013	Caswell et al. (2020) (69)
Asia	Bangladesh	Dietary survey sampled based on response to agricultural intervention (WDDP Bangladesh 1)	412	Women	Subsample	2	24HR	Total energy, vitamin A, thiamin, vitamin B-12, folate, iron, zinc	1996	Arimond et al. (2009) (70, 71)^8^
Asia	Bangladesh	Multistage cluster survey in two rural regions (WDDP Bangladesh 2)	926	Women, children	All or most	2	Combination	Total energy, vitamin A, thiamin, vitamin B-12, folate, iron, zinc, phytate	2007–2008	Arsenault et al. (2013) (21, 82)^8^
Asia	Philippines	Two-stage cluster community survey (2005 Cebu Longitudinal Health and Nutrition Survey, WDDP)	2045	Women	All or most	2	24HR	Total energy, vitamin A, thiamin, vitamin B-12, folate, iron, zinc	2005	Daniels et al. (2009) (72, 73)^8^
Asia	Philippines	Observational study sampled based on levels of vitamin A intake (GloVitAS-P)	123	Children	All or most	2	24HR	Total energy, vitamin A, retinol	2016–2017	Ford et al. (2020) (83)
Caribbean	Puerto Rico	FFQ validation study	242	Children	All or most	2	24HR	Total energy, vitamin A, retinol, thiamin, vitamin B-12, folate, folic acid, iron, zinc	2014–2015	Palacios et al. (2017) (74)
Central America	Guatemala	Randomized controlled trial of multiple micronutrient supplements with bovine serum concentrate (longitudinal data)	259	Children	All or most	2–4	Combination	Total energy, vitamin A, retinol, thiamin, vitamin B-12, folate, iron, zinc, phytate	1997–1999	Bégin et al. (2008) (84)
Europe	Sweden	FFQ validation study within randomized controlled trial (MINISTOP)	40	Children	All or most	Up to 4	24HR	Total energy, vitamin A, retinol, thiamin, vitamin B-12, folate, iron, zinc	2014–2015	Nyström et al. (2017) (75)

1 CSFII, Continuing Survey of Food Intakes by Individuals; EPIC, European Prospective Investigation into Cancer and Nutrition; FFQ, food-frequency questionnaire; GloVitAS-P, Global Vitamin A Safety Assessment Study–Philippines; MINISTOP, Mobile-based Intervention Intended to Stop Obesity in Preschoolers; NR, not reported; WDDP, Women's Dietary Diversity Project; 24HR, 24-h dietary recall.

2 Total sample sizes refer to all combined subgroups and do not necessarily represent sample sizes per analytical unit.

3 The number of days of dietary intake data among those with repeats is presented as an average or range, unless otherwise noted.

4 Listed nutrients include those for which data were extracted for the present study, although other nutrients may have been reported in the publication.

5 Nyambose et al. note in their publication that the variance ratios from this study were unpublished data obtained via a personal communication with Suzanne Murphy, Cancer Research Center of Hawaii, University of Hawaii at Monoa.

6 Full text was not accessible to the authors.

7 Median.

8 Original dataset is publicly available online; see reference for access information.

Among NPNL WRA, WIV:total appears to vary among studies from different countries, although insufficient data were available to distinguish between country and study design differences. Within 3 countries for which estimates were available for both rural and urban and/or mixed settings for this subpopulation (42, 67,72, 76, 77, 79), a pattern of lower ratios in rural compared with other settings was noted for energy, thiamin, vitamin B-12, zinc, and iron intakes in Burkina Faso and the Philippines, as well as in Uganda for iron and zinc. In 1 dataset from a study in Burkina Faso (77), disaggregated analyses for 2 seasons, lean and postharvest, revealed that the proportion of variance coming from WIV appears to change across seasons for some nutrients, although there was no distinct pattern of increasing or decreasing across seasons.

A sensitivity analysis on the impact of covariates on variance components using nationally representative survey data from Cameroon (19) revealed that in many (but not all) cases, adding covariates to the model led to increases in WIV:total (differences from −0.12 to +0.24), which was largely explained by reductions in BIV in adjusted models (data not shown).

Complete variance ratio data from publications and reanalyzed datasets, along with population and study characteristics, are available at https://osf.io/wyvug/.

### Simulated effect of varying the within-person variance component on prevalence of inadequate intake

**FIGURE 3:**
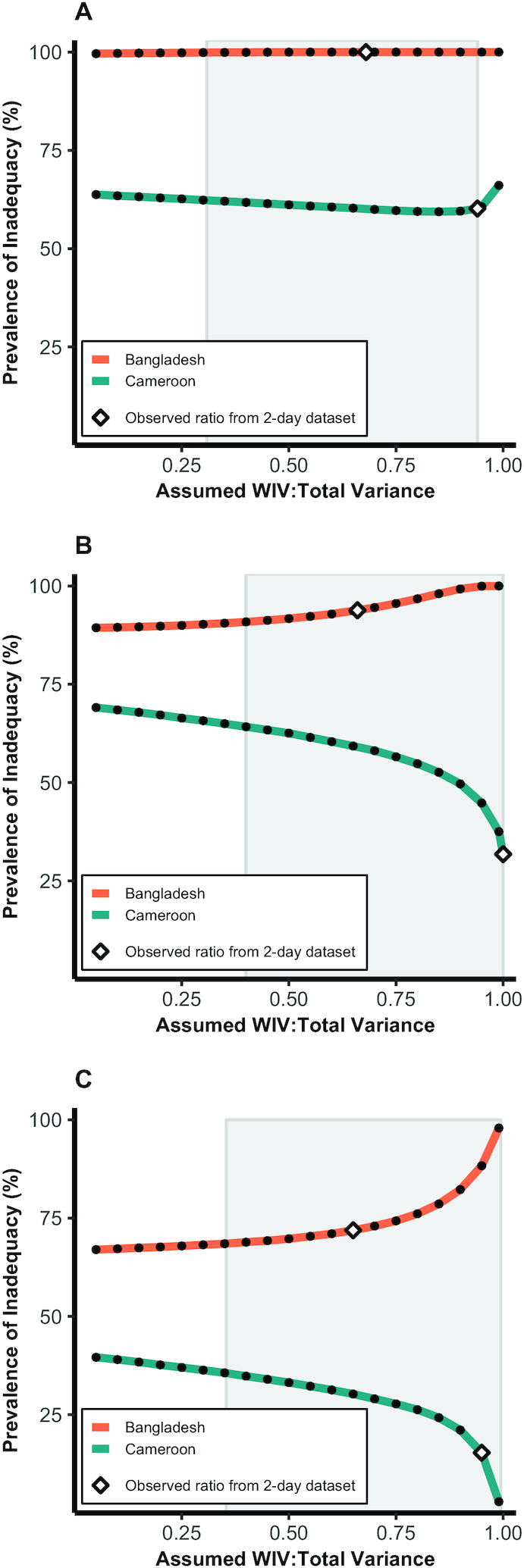
Simulated effect of varying the ratio of within-individual to total variance on the prevalence of folate (A), vitamin A (B), and zinc (C) inadequacy among women of reproductive age in Cameroon and Bangladesh using the 1-d NCI method. Prevalence using WIV:total calculated from 2-d data on the same population is marked by diamonds. The range of WIV:total observed in existing datasets among NPNL or mixed (NPNL and lactating/pregnant) populations is indicated by shaded boxes. NCI, National Cancer Institute; NPNL, nonpregnant and nonlactating; WIV:total variance, ratio of within-individual to total variance.

**Figure 3** shows the simulated effect of varying WIV:total on the prevalence of inadequate intake of folate, vitamin A, and zinc using data from Bangladesh and Cameroon, in comparison to the prevalence estimated using WIV:total ratios from 2-d data from the same study and in relation to the range of values reported in this paper for WRA (shaded region). The latter represents the range from which a researcher might select an external variance ratio. The effect of the choice of WIV:total on estimated prevalence varied by population and nutrient. For example, there was little effect of changing WIV:total on prevalence of folate inadequacy. In contrast, more notable shifts were observed in the case of zinc, for which estimated prevalence of inadequacy in Cameroon dropped from 39.6% when WIV:total was set to 0.05 to 2.9% when WIV:total was assumed to be 0.99. Within the simulations corresponding to values of WIV:total found in existing datasets (shaded region), deviation from the observed ratio from the full dataset resulted in a difference in estimated prevalence of up to 32.4 percentage points (vitamin A in Cameroon). The magnitude of the effect appeared to be greatest at WIV:total ratios >0.95, except in the case of vitamin A in Bangladesh.

**FIGURE 4:**
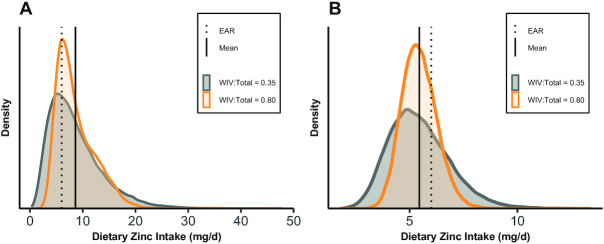
Modeled distributions of usual zinc intake in women in Cameroon (A) and Bangladesh (B) assuming a low within-individual to total variance ratio (WIV:total = 0.35) or high ratio (WIV:total = 0.80) using the 1-d NCI method. The EAR for zinc of 6 mg/d for nonpregnant, nonlactating women assuming a mixed or refined vegetarian diet [as determined by the International Zinc Nutrition Consultative Group (24)] was used as the cutoff for inadequacy. EAR, Estimated Average Requirement; NCI, National Cancer Institute; WIV:total, ratio of within-individual to total variance.

The direction of the effect of varying WIV:total on prevalence of inadequacy differed between the 2 populations in this analysis. This difference can be explained by the shape and position of the distributions in relation to the EAR, which is demonstrated using the example of zinc in **Figure 4**. The mean usual zinc intake in Cameroon (solid line, panel A) is greater than the EAR (dotted line), while in Bangladesh the opposite is true. As the assumed proportion of variance from WIV (i.e., the amount of variance removed from the distribution during adjustment) increases, the modeled variance of usual nutrient intakes decreases. In the case of the Cameroon data, this resulted in decreased estimated prevalence of inadequacy (approaching 0%), while for Bangladesh the adjustment resulted in increased prevalence (approaching 100%). In sensitivity analyses, SEs of inadequacy prevalence generally increased with increasing ratios (data not shown).

## Discussion

We compiled variance component ratios from a range of age and sex groups in diverse rural and urban settings, with a focus on increasing the availability of variance ratios from low- and middle-income countries. Some patterns emerged by nutrient, age and sex (especially among children), and setting, but in general, we observed wide variation in values, even within nutrients and subpopulations. Simulation analyses demonstrated that the assumed WIV:total ratio can affect estimated usual intake distributions and prevalence of inadequate intake, but not in a consistent way—that is, the extent and direction can vary across nutrients and populations depending on characteristics of the distribution and its position in relation to the EAR.

Variance ratios have previously been compiled and published from single studies, such as NHANES (62), or small collections of studies (35, 38, 40, 47, 57). Here we provide data on variance ratios of nutrient intakes from a wide range of populations and geographic contexts. While efforts were made to include a wide array of variance ratio data, a formal systematic review was not undertaken in this study, and some published variance ratio estimates may have been missed. Further, the scope of this study was limited to a selected list of nutrients, which, in general, were chosen for their public health importance; however, some nutrients of public health importance, such as iodine and calcium, were not included. Nevertheless, these data can be expanded upon as additional estimates become available and may be useful for future studies to increase understanding of how variance ratios vary in different contexts. The database can also serve as a resource for selecting an external variance ratio when analyzing single-day dietary data. However, users must exercise caution due to varying analytical techniques and reporting methods, the potential for differences between the external study population and the target study population, and the substantial impact selecting the incorrect variance ratio can have on results. Recommendations for how to approach selecting an external estimate are detailed in a later section.

### Explaining variation in the WIV:total variance

A number of factors can influence food intake patterns and diversity, thereby affecting ratios of WIV:total variance in nutrient intakes through influences on WIV, BIV, or both. It is fairly well established that variance ratios vary by nutrient (34, 60, 64, 90). In addition, variance components are often calculated for specific age, sex, and life-stage groups, in accordance with nutrient adequacy studies that require stratified analyses to account for different nutrient requirements among these groups. Previous observations on differential variance components and ratios by age and sex have been made, although differences in variance ratios were not usually compared statistically. Among these observations, researchers have noted inconsistent patterns (45, 47) or no substantial difference (65) in variance ratios by sex but have observed some variation among age groups, especially in children (34, 45, 47, 65). In particular, lower ratios were found for very young children (2.5- to 3-y-olds) compared with older children (4- to 6.5-y-olds) (45), similar to the pattern of lower variance ratios for children <1 y of age observed in this study (Figure 2). Low WIV in this age range, especially during infancy, is expected since the diet of an infant in the first few months of life consists primarily of 1 food source (breast milk or formula), followed by the addition of specific complementary foods that may be limited in variety initially. Conversely, the diets of older children and adults are more complex and responsive to a range of influences (91–93), and therefore may have higher day-to-day variation.

In this study, substantial variation existed in WIV:total across studies from different geographic settings, in agreement with previous research. For example, a study comparing nutrient intake variance components and ratios among children from Russia and the United States observed generally lower ratios in Russia (47). Differences in variance ratios were also found between 2 regions in India (36). Plausible explanations for these differences include distinct culinary traditions and preferences, religious and cultural practices, food production systems, and socioeconomic contexts. However, apart from 1 study that found statistically different variance ratios of energy and protein intakes among 5 income groups (28), direct evidence of the effect of these factors on WIV or WIV:total appears to be scarce. Nevertheless, observed variation in dietary intakes by factors such as socioeconomic status (94), religious fasting (95), and rural versus urban setting (96, 97) plausibly influences variance ratios as well. This is partially supported by our observation that, within the few countries for which comparisons were available, variance ratios tended to be higher in urban compared with rural settings. Additional research is needed to confirm the contribution of these and other environment-level factors to WIV and BIV in nutrient intakes.

In addition to the aforementioned factors that may influence true day-to-day variation in nutrient intakes, the datasets and publications included here varied in a number of study design–related factors, including the dietary assessment method used (i.e., observation vs. recall, portion-size estimation, recipe data collection, etc.), sample size, number of days of dietary intake data per individual, and time interval between dietary measurements. The potential composite effects of these factors on variance ratios are difficult to discern. In 2 studies comparing observed weighed food records with 24HRs collected for the same days, variance ratios from weighed food records were observed to be lower (98) or different but in an inconsistent direction across nutrients (43). In the current analysis, disparities in variance component ratios among women in rural Bangladesh were found between 2 datasets (21, 70) that used different dietary assessment methods (e.g., WIV:total of folate intake was 0.68 from weighed food records vs. 0.91 from 24HRs), although differences in the regions studied, the survey year, or other factors could have contributed to the variation. Intake data collected on consecutive days versus nonconsecutive days can result in reduced WIV due to autocorrelation among consecutive days of intake (44), while increasing the sample size and number of days of intake data would be expected to result in more accurate estimates, although an upper threshold on such benefits is likely (99). The timing of data collection may also be important in some contexts, since nutrient intakes can vary across season in settings in which the food environment is dependent on local agriculture and/or availability of wild foods (69, 77, 100–102). Correspondingly, lower WIV:BIV ratios during harvest versus non–harvest seasons were reported for some nutrients in a study of pregnant women in rural Malawi (38). Previously reported seasonal differences in nutrient intakes among women and children in Burkina Faso (77) appeared to translate into shifts in the WIV:total ratios in the present study. Beyond seasonal effects, WIV:total ratios for a given nutrient, subpopulation, and geography could shift over time due to changing food systems and subsequent consumption patterns, or due to evolving survey methodologies. Although differences in WIV:total were relatively small across survey years in nationally representative surveys in Russia and the United States (7, 8, 47), more research is needed to understand how WIV:total ratios might shift amidst rapidly changing food landscapes, especially in low- and middle-income countries.

Finally, authors of the reviewed studies used a number of different statistical approaches for calculating variance components of nutrient intake. In particular, as we demonstrated in sensitivity analyses, the inclusion of various covariates can have a substantial effect on the variance ratio by reducing the overall residual variance (BIV + WIV), with most of the reduction being attributed to BIV. Given the difficulty in comparing across studies, we generated new estimates from existing datasets using a consistent calculation procedure to control for statistical method. Nevertheless, other sources of variation in study designs remained, and wide variation was still found in the variance ratios using this approach. Multicenter studies using a consistent study design and analytical approaches across study sites would allow for more robust examination of true variation in WIV:total of nutrient intakes across geographic contexts.

### Impact of the WIV:total estimate on prevalence of inadequate nutrient intakes

In our simulations, varying WIV:total incrementally from 0.05 to 0.99 changed the estimated usual nutrient intake distributions and, consequently, the estimated prevalence of inadequate intake in most cases examined. While this effect is to be expected given the mathematical procedure used to produce the model, the inconsistency of the extent and direction of this effect across the 3 nutrients and 2 populations studied suggests that characteristics of the dataset and how it is modeled may result in different effects of WIV adjustment on prevalence of inadequacy. In particular, the position of the distribution in relation to the EAR, in cases where the EAR cut-point method is used, would be expected to determine whether prevalence of inadequacy increases or decreases with changes in WIV:total, as we observed here (Figure 4). Our simulations also displayed more pronounced changes in modeled prevalence at very high WIV:total. However, it is important to note that, while WIV:total ratios of up to 0.99 were included in our simulations to mirror the range of values found in existing studies, models of usual nutrient distributions can become invalid as WIV:total approaches 1, since they may result in distributions with implausibly low variances and/or violations of model assumptions (e.g., the modeled variance in usual intakes could be reduced, through overadjustment, to less than the variance in nutrient requirements). An extreme example of this is when WIV:total is “equal” to 1.0, which would result in all variance being removed from the model, returning a single point estimate rather than a distribution. Additionally, wider CIs around higher variance ratio estimates have been reported (44); therefore, WIV:total estimates approaching 1.0 are likely imprecise and may introduce further uncertainty into resulting models.

Several other studies have conducted sensitivity analyses on the robustness of estimated prevalence of inadequate nutrient intakes to the variance ratio assumption (7–9, 35). Jahns et al. (7) evaluated the use of external adjustment factors, repeat measures, or no adjustment to model distributions of vitamin C usual intake in Russian children and found only small differences (2.6–4.3 percentage points) in the prevalence of inadequate intake when a US-derived WIV:total ratio was used, which greatly reduced the amount of bias introduced compared with no adjustment. Sensitivity analyses conducted by Gibbs et al. (35) found that the use of an external variance estimate greatly overestimated (by 19 percentage points) the prevalence of inadequate calcium intakes in Filipino infants but had little to no effect on prevalence estimates for Filipino toddlers or on prevalence of iron inadequacy in either age group. Notably, the external ratio applied to the infant data in this case was derived from data on toddlers, highlighting the potential importance of correctly matching by age in children. More recently, Luo et al. (9) assessed the effect of applying biased external variance ratios to the NCI 1-d method to estimate usual nutrient intake distributions using NHANES 2011–2014 data on intakes of 4 nutrients (vitamin A, magnesium, folate, and vitamin E) among men. The authors observed divergent patterns by nutrient in the effect of mis-specifying the variance ratio on estimated prevalence of inadequate intake, both in terms of the magnitude and direction of the effect (9). The present analysis builds on these observations by examining a different population (women) and nutrient (zinc) and highlights that divergent effects may also appear among analyses of the same nutrient and life-stage group when the distributions of intakes differ between populations.

The results suggest that researchers using external variance ratios cannot assume a consistent direction of bias and must conduct sensitivity analyses with their dataset. In accordance with previous research, the present analysis highlights the importance of conducting sensitivity analyses to determine the robustness of prevalence estimates to varying WIV:total in single-day dietary studies. Researchers and policy makers must then determine what level of uncertainty is acceptable for their intended use of the data. For example, if a governing body is interested only in whether there is likely a high or low prevalence of inadequate intake of a given nutrient in the population, a somewhat higher degree of uncertainty may be acceptable. If, however, the aim is to track potentially small changes in prevalence from year to year to determine, for instance, the effectiveness of a fortification program, more precise estimates may be needed. Imprecise prevalence estimates resulting from the use of biased variance ratios could influence policy decisions if prevalence is wavering near a cutoff being used to determine whether a public health problem exists. For example, in our simulations of zinc inadequacy in Cameroon, altering the assumed WIV:total ratio produced a range of estimated prevalence that included a cutoff (>25%) previously used as an indicator pointing to a public health problem (103).

### Guidance for researchers

Our research group has previously published a method for analysis of single-day dietary data (9). This paper complements previous work by providing guidance on selection of an external variance ratio. These tools are meant to improve analyses of single-day data. However, we strongly urge researchers planning new studies not to resort to this method. Given wide variation in WIV:total across studies, potential sensitivity of ratios to methodological and study design factors, and uncertainty surrounding the stability of usual nutrient intake distributions and prevalence estimates to variation in the assumed ratio, the collection of ≥2 d of intake data in at least a representative subsample in dietary studies of populations is strongly encouraged. Moreover, even when repeat recalls are collected, it is possible for the statistical model to produce implausibly high variance ratios (e.g., WIV:total near or at 1.0), as seen in several instances in this literature review and analysis. In such cases, researchers may consider data aggregation or other approaches to improve variance estimates, where possible, or selection of an external estimate from a similar population using the same procedure described below for single-day datasets.

For researchers intending to analyze single-day data, we offer the following guidelines for choosing an external variance ratio to minimize the effects of bias associated with their use.

#### Match the reference value as closely as possible by nutrient, population, study design, and analytical approach

Reference studies should ideally match the nutrient of interest and the target population based on sex and life stage. The latter is particularly important in the case of very young children. Physiological status among women (i.e., pregnancy and lactation) might also be important in some cases, although more research is needed. It also may be important to consider social and environmental factors that are similar or different between the reference and primary studies (including factors that vary over time within a population), and how these factors may be more or less relevant to dietary behaviors and resultant nutrient intake patterns in the studied populations. Based on our and other researchers’ work, some such factors that appear to influence variance ratios include country (47) or region (36), income level (28), urban versus rural setting, and the season during which the data were collected (38). It is also theoretically advantageous to consider other factors, such as food security and cultural and religious traditions, although we did not identify data specifically examining these topics.

Since differences in the study design may lead to discrepant variance ratios, reference studies with a similar study design to the primary study and of acceptable quality to provide robust variance estimates should be selected. Study design factors to consider include the dietary assessment method, sampling procedure, total sample size, number of individuals with repeat observations, number of observations per individual, and the time between observations. While our results do not allow conclusions about whether 1 dietary assessment method would be preferable for application of external ratios, we note that there are far fewer variance ratios available from studies using observed records; thus, it may be challenging to match by study population and data collection method if the data to be analyzed include food records. Repeated dietary observations should ideally be performed on nonconsecutive days to minimize correlation of intake on consecutive days; if consecutive days are used, a minimum of 3 days is recommended (3). Although it is generally accepted that 2 nonconsecutive days of intake on at least a subset are sufficient to estimate variance components and a population distribution through statistical modeling, unlike the higher numbers needed to estimate usual nutrient intakes of individuals (59, 104), more research is warranted on whether estimates of population distributions could have improved precision with more days, particularly for nutrients with high WIV. With regard to sample sizes, a minimum of 100 individuals per subgroup, including at least 40–50 individuals with a second observation, has recently been recommended for adjusting usual zinc intake distributions for WIV (105), while the Institute of Medicine (IOM) previously recommended at least 30–40 individuals with replicates in dietary surveys (3). These recommendations may serve as a useful rule of thumb in selecting a reference study, although more research on minimum sample sizes for calculating variance components from dietary data is needed, and new studies should perform calculations to determine the required sample size to match their analytical goals (106). {The statistical rationale for the aforementioned recommendations is not well documented, and given the IOM recommendations were made with the Iowa State University method in mind, they may not be generalizable to other methods of variance component estimation. In our reanalysis of datasets, we used a mixed-effects [i.e. multilevel] model for estimation of variance components, so simulation studies of multilevel models using maximum likelihood estimation from other fields may provide insight into required sample sizes. Such studies have led to recommendations for the minimum number of “clusters” [which would translate to number of individuals in repeated dietary recall studies] of 10 (107), 30 (108), and 100 [reviewed in (107)] to accurately estimate variance components [not considering SEs]. However, these studies used cluster sizes of ≥5, which may limit their generalizability to dietary surveys, where cluster sizes [i.e. days of data per individual] of 2 are commonly used.}

Finally, the comparability of the statistical approach used to produce the variance estimates in the reference study in relation to the primary analysis to be conducted should be considered, since different statistical methods can produce different estimations of WIV and BIV and their ratio. In particular, the inclusion of influential covariates can account for some of the variation between individuals, often leaving less residual BIV and resulting in higher WIV:total. Thus, it is advisable to choose reference studies that included covariates similar to those that will be used in the primary analysis.

#### Exclude implausible WIV:total values

If a reasonable number of criteria are met, reference values presented here or in other publications may be considered as external variance ratios. Alternatively, new variance ratios can be calculated if the original data on a comparable reference population is accessible. The NCI MIXTRAN macro (20), PC Software for Intake Distribution Estimation (PC-SIDE) (109), or the analytical code provided in this study represent a few methods by which variance components may be calculated for intakes of foods and nutrients consumed daily or nearly daily. In either case, extremely high WIV:total values should not be considered for use as external adjustment factors in analyses of single-day dietary data, given their likely implausibility and potential to decrease the validity of statistical models. A cutoff of WIV:BIV >10 (WIV:total >0.91) has previously been used to identify inappropriately high variance ratios (58). We further recommend that researchers always visualize modeled distributions to assess the plausibility of the model, even at lower variance ratios. In the case of new variance component analyses, aggregated analyses of age groups to increase the sample size, if appropriate, and/or outlier removal procedures may result in more robust variance ratios (58).

#### Conduct sensitivity analyses

Once an external variance ratio has been selected, this value may be applied to the NCI 1-d method (for nutrients consumed nearly daily) or other methods for estimating the usual intake distribution. Given the range of factors potentially influencing variance ratios and the difficulty in predicting variation across studies, sensitivity analyses should be conducted in all cases to determine the robustness of prevalence estimates to changes in the WIV:total or WIV:BIV ratio for a particular dataset. The range of variance ratios tested should represent the uncertainty in the external ratio estimate, considering the available estimates from similar populations that meet the aforementioned criteria. If such a range is unavailable, we recommend testing a range of WIV:total values from 0.5 to 0.9, or wider, for populations >1 y of age. If a high degree of uncertainty exists, it may be more appropriate to present the study results as a range of possible prevalence rather than a point estimate. SEs for each prevalence estimate should be calculated to determine changes in the precision of estimates.

### Conclusions

When external estimates of within- and between-person variation in nutrient intakes are used in lieu of collecting multiple days of dietary intake data, a high degree of uncertainty is introduced into resulting models of usual nutrient intake distributions. We discovered wide variation in WIV:total ratios across studies and observed few consistent patterns due to the large number of potentially influencing variables, suggesting this ratio is difficult to accurately predict from external studies given its sensitivity to a number of factors specific to the population of interest, study design, and statistical approach. The effect of improperly choosing the variance ratio on estimated prevalence of inadequacy is dataset-specific and can be substantial (up to 32 percentage points in the datasets studied); therefore, even when applying the suggested procedure to select an external variance estimate, prevalence estimates should be assessed and reported using sensitivity analyses around credible ranges of WIV:total to quantify ranges of uncertainty. Studies in the planning stage should prioritize the collection of multiple days of dietary data if the prevalence of the population at risk of inadequate or excessive intakes is of interest.

## Notes

Supported by the Bill & Melinda Gates Foundation. Role of funder: A member of the VCNI Data Working Group, KH Brown, was employed by the BMGF at the time this grant was awarded, but he had no involvement in decision making regarding the award or subsequent management of the project by the foundation.

Author disclosures: The authors report no conflicts of interest.

Members of the Variance Components of Nutrient Intakes (VCNI) Data Working Group of the Micronutrient Intervention Modeling (MINIMOD) project are listed in the Acknowledgments section.

Abbreviations used: BIV, between-individual variation; EAR, Estimated Average Requirement; IOM, Institute of Medicine; NCI, National Cancer Institute; NPNL, nonpregnant, nonlactating; WIV, within-individual variation; WRA, women of reproductive age; 24HR, 24-h dietary recall.
